# 973. Occupational Therapy Protocol Following Application of Biodegradable Temporizing Matrix to Bilateral Hand Burns

**DOI:** 10.1093/jbcr/irag033.570

**Published:** 2026-04-06

**Authors:** Catherine C Greer

**Affiliations:** Regional One Health Firefighters Burn Center, Memphis, TN

## Abstract

**Introduction:**

The purpose of this retrospective case series was to review outcomes of a specialized OT protocol following application of BTM to three patients with full thickness burns to their bilateral hands. The focal points of this protocol included early compression management and aggressive elongation techniques carried out by trained occupational burn therapists at a single burn center.

**Methods:**

This single center retrospective case series utilized electronic medical records review to assess patients admitted from December 2024 to March 2025 who received BTM to at least one hand. Inclusion criteria for this study included patients 18 years of age or older; ^2^full thickness burns to at least one hand; ^3^BTM applied to at least one hand; ^4^and received burn specific occupational therapy intervention. Therapy interventions and outcomes were collected, including treatment plan, functional status post BTM and functional status following discharge from burn center.

**Results:**

As a result, 67% of patients demonstrated positive short- and long-term outcomes related to edema reduction, desensitization, and full hand function bilaterally. 33% of patients were documented as having less than full functionality of at least one hand due to scar tissue development, but favorable results regarding presence of edema and tactile sensitivity. None of the patients reviewed experienced long-term disability or the inability to return to pre-burn activity levels.

**Conclusions:**

Based on these results, it was determined that implementation of a specialized OT protocol is necessary and effective in bringing about positive short- and long-term outcomes related to a burn patient’s care.

**Applicability of Research to Practice:**

The use of this protocol including coban wrap post operatively and early mobilization techniques demonstrated an improvement in functional outcomes related to bilateral hand function.

**Funding for the study:**

N/A.

